# Growth under Different Trophic Regimes and Synchronization of the Red Microalga *Galdieria sulphuraria*

**DOI:** 10.3390/biom11070939

**Published:** 2021-06-24

**Authors:** Vít Náhlík, Vilém Zachleder, Mária Čížková, Kateřina Bišová, Anjali Singh, Dana Mezricky, Tomáš Řezanka, Milada Vítová

**Affiliations:** 1 Centre Algatech, Laboratory of Cell Cycles of Algae, Institute of Microbiology of the Czech Academy of Sciences, Novohradská 237, 379 81 Třeboň, Czech Republic; nahlik@alga.cz (V.N.); zachleder@alga.cz (V.Z.); cizkova@alga.cz (M.Č.); bisova@alga.cz (K.B.); singh@alga.cz (A.S.); 2 Faculty of Science, University of South Bohemia, Branišovská 1760, 370 05 České Budějovice, Czech Republic; 3 Department of Medical and Pharmaceutical Biotechnology, IMC University of Applied Sciences, Piaristengasse 1, 3500 Krems, Austria; dana.mezricky@fh-krems.ac.at; 4 Institute of Microbiology of the Czech Academy of Sciences, Vídeňská 1083, 142 20 Prague, Czech Republic; rezanka@biomed.cas.cz

**Keywords:** cell cycle, red algae, *Galdieria*, growth, cell division, light intensity, temperature, trophic regimes, synchronization

## Abstract

The extremophilic unicellular red microalga *Galdieria sulphuraria* (Cyanidiophyceae) is able to grow autotrophically, or mixo- and heterotrophically with 1% glycerol as a carbon source. The alga divides by multiple fission into more than two cells within one cell cycle. The optimal conditions of light, temperature and pH (500 µmol photons m^−2^ s^−1^, 40 °C, and pH 3; respectively) for the strain *Galdieria sulphuraria* (Galdieri) Merola 002 were determined as a basis for synchronization experiments. For synchronization, the specific light/dark cycle, 16/8 h was identified as the precondition for investigating the cell cycle. The alga was successfully synchronized and the cell cycle was evaluated. *G. sulphuraria* attained two commitment points with midpoints at 10 and 13 h of the cell cycle, leading to two nuclear divisions, followed subsequently by division into four daughter cells. The daughter cells stayed in the mother cell wall until the beginning of the next light phase, when they were released. Accumulation of glycogen throughout the cell cycle was also described. The findings presented here bring a new contribution to our general understanding of the cell cycle in cyanidialean red algae, and specifically of the biotechnologically important species *G. sulphuraria*.

## 1. Introduction

The unicellular red alga, *Galdieria sulphuraria,* belongs to Cyanidiophyceae, the class of primitive rhodophytes, which includes the genera *Cyanidioschyzon*, *Cyanidium* and *Galdieria* [1]. The genomes of three representatives of cyanidiales have been sequenced so far—*Cyanidioschyzon merolae*, *G. sulphuraria* and *Galdieria phlegrea* [2,3,4,5]. These extremophilic microalgae are adapted to thermo-acidophilic growth conditions, inhabiting hot sulfur springs and geothermal habitats [6,7,8]. They thrive in a wide range of temperatures up to 56 °C and in acidic environments with pH values below 1.0 [9,10]. The adaptation of cyanidialean red algae to high temperature and low pH [9,11] and their resistance to high salt [12], numerous toxic metals [13,14], as well as rare earth elements (REEs) [15,16] is unusual among other eukaryotic algae and makes it a promising candidate for biotechnological exploitation [17,18]. *Galdieria* is not only resistant to high levels of metals and rare earths, but it can also accumulate them inside the cells [14,15,16,19]. *G. sulphuraria* is usually cultivated for several biotechnological applications such as recycling of valuable components and nutrients from low pH wastewaters; production of phycocyanin as a main pigment even under heterotrophic conditions; cultivation for biofuels, due to its high resistance to contamination, even under heterotrophic conditions; production of glycogen; nutritional applications (reviewed in [17]). Unlike other Cyanidiophyceae, *G. sulphuraria* shows distinguished metabolic versatility which, apart from autotrophic growth, permits mixotrophic and heterotrophic growth on a variety of carbon sources [20,21,22]. One of the organic carbon sources being utilized by *Galdieria* is glycerol [23], which is produced in enormous amounts as waste during biodiesel production, making it the cheapest organic carbon source for microbial cultivation [24,25]. Under all trophic conditions, the primary carbon and energy storage compound of *G. sulphuraria* is glycogen. This is common for red algae but distinct from green algae and higher plants [26]. Glycogen of *G. sulphuraria* is typically highly branched and of a low molecular weight [27].

In autotrophically grown algae, two abiotic factors set the growth rate: light intensity (and duration) [28,29,30] and temperature [31,32,33]. High light intensity generally accelerates growth and photosynthesis of algae [34], and the production of more daughter cells within one multiple fission cell cycle (for details see below). Changes in temperature affect the entire metabolism, including, but not limited, to growth rate, nutrient uptake, CO_2_ sequestration, and the chemical composition of the algae [35]. Furthermore, the duration of the cell cycle and critical cell size are affected by temperature, independent of light intensity [30,36,37,38]. A temperature increase of 10 °C causes an up to two-fold increase in metabolic rate [33,37], although, if the temperature increases above the threshold, it will cause the arrest of cell division [31,37,39] or even a decrease in growth rate [40]. The optimal temperature for growth of *G. sulphuraria* is 40–42 °C. Although, it can grow over a wide range of temperatures from 20 °C to 56 °C [10,18]. For *G. sulphuraria*, pH is another important abiotic factor affecting its growth. Being acidophilic, it thrives over a pH range from 0.05–5.0. Changes in pH significantly affect growth and chemical composition, particularly the lipidomic profile, of *G. sulphuraria* [11,23,41].

Similar to some other microalgae, *G. sulphuraria* divides by multiple fission into four, or up to 32 daughter cells [1,42,43]. Multiple fission leads to the release of generally 2^n^ daughter cells from a single mother cell within one cell cycle. The cell cycle includes processes of cell growth (growth sequence—G1 phase) and reproduction (reproductive sequence—replication and nuclear division—S and M phases, respectively). The two sequences are connected by cell division. Multiple growth and reproductive sequences overlap in the multiple fission cell cycle. Each growth phase leads to an approximate doubling of cell size, cell mass, bulk RNA, and bulk protein per cell (for a review, see [44,45]). Doubling, or attaining a critical cell size, seems to be a pre-requisite for entry into the cell cycle at commitment point (CP), which is the functional equivalent to START in yeasts [46] and the restriction point in mammalian cells [47]. There are several ways to organize the reproductive sequences, and these translate into different patterns of division. The two basic patterns are consecutive (Scenedesmus pattern) and clustered (Chlamydomonas pattern) [45]. In the Scenedesmus pattern, each reproductive sequence is organized very similar to the standard cell cycle of binary fission, i.e., entry into reproductive sequence at CP is followed by DNA replication (S phase), G2 phase, M phase and cell division. Such reproductive sequences overlap within a single multiple fission cycle (for review see [44,45]). In contrast, the Chlamydomonas pattern is characterized by a long gap phase followed by several rounds of alternating S and M phases and it seemingly lacks any G2 phase (for review see [44,45,48]). Apart from the two extremes, there are different combinations of reproductive sequences, such as in the cell cycles of *Haematococcus pluvialis* [49] or *Parachlorella kessleri* [50].

Cultures of algae are routinely synchronized by alternating light/dark (L/D) regimes, which mimic situation in nature. When grown autotrophically, the cells cannot grow in dark but are able to divide. Therefore, they all start growing exactly at the same life stage and time resulting in a synchronous population [45].

While the methodology for synchronization of green algae is well-developed [51], synchronization of red microalgae remains limited. Among the specialized extremophilic relatives of *G. sulphuraria*, only one species was studied extensively, *C. merolae* dividing by binary fission with the S and M phases occurring in the dark. It was synchronized successfully by an alternating light/dark (L/D) regime [52] and its cell cycle, particularly the coordination between mitotic and organelle divisions, have been studied extensively [53,54,55,56,57,58]. The cell cycle of the mesophilic unicellular red alga *Porphyridium* sp. was studied [59,60] and synchronization by L/D regime were reported for *Porphyridium purpureum* [61,62] and *Porphyridium cruentum* [63]. Information on the cell cycle of *G. sulphuraria* remains limited. Recently, *G. sulphuraria* strain 074W was partially synchronized by a L/D regime of 12/12 h under low light conditions (100 μmol photons m^−2^ s^−1^). This kind of treatment has led to the enrichment of dividing cells in the dark period, but the percentage of dividing cells within the population was quite low, 10–15% [43]. This treatment allowed microscopic analysis of the number of daughter cells formed and demonstrated the existence of critical cell size at CP, but it did not permit a more thorough analysis of cell cycle progression.

In the present study, we followed the effect of different parameters—pH, light intensity, temperature, and trophic regimes—on the growth of *G. sulphuraria*. For the mixotrophic regime, glycerol was used as a cheap organic carbon source. We synchronized the culture of *G. sulphuraria* under optimal growth conditions by L/D cycle and characterized the timing of CP attainment, DNA replication, nuclear and cellular division(s). Furthermore, we analyzed the glycogen accumulation profile of synchronously growing and dividing cultures.

## 2. Materials and Methods

### 2.1. Organism and Culturing

The unicellular red alga *Galdieria sulphuraria* (Galdieri) Merola, 002 was obtained from the Algal Collection of Dipartimento delle Scienze Biologiche, Section of Plant Biology, University “Federico II” of Naples, Italy. The algae were grown in modified Galdieria-nutrient medium [11], pH 1.5–4 adjusted with HNO_3_, to the following final composition of macroelements (g L^−1^): 1.31 (NH_4_)_2_SO_4_, 0.27 KH_2_PO_4_, 0.25 MgSO_4_·7H_2_O, 0.02 C_10_H_12_O_8_N_2_NaFe, 0.14 CaCl_2_·2H_2_O, and microelements diluted 500x from the stock solution (mg L^−1^): 31 H_3_BO_3_, 1.25 CuSO_4_·5H_2_O, 22.3 Mn SO_4_·4H_2_O, 0.88 (NH_4_)_6_Mo_7_O_24_·4H_2_O, 2.87 ZnSO_4_·7H_2_O, 1.46 Co(NO_3_)_2_·6H_2_O, 0.014 V_2_O_4_(SO_4_)_3_·16H_2_O, 0.3 Na_2_NO_4_·7H_2_O, 1.19 KBr, 0.83 KI, 0.91 CdCl_2_, 0.78 NiSO_4_, 0.12 CrO_3_, 4.74 Al_2_(SO_4_)_3_K_2_SO_4_·24H_2_O (all chemicals from Penta, Chrudim, Czech Republic) in distilled water, autoclaved for 20 min. Glycerol (to a final concentration of 1% (*v*/*v*)) (Penta, Chrudim, Czech Republic) was added as a source of energy and carbon for the mixotrophic cultivations [16]. The algae were routinely sub-cultured on culture plates containing Galdieria-nutrient medium solidified with 3% agar every three weeks.

The cultures were grown in glass cylinders (300 mL) or flat (2.5 L) photobioreactors. The cylinders or photobioreactors were placed in a thermostatic water bath and illuminated from one side by a panel of dimmable fluorescent lamps (OSRAM DULUX L55 W/950 Daylight, Milano, Italy) allowing adjustment of the incident light intensity from 16 to 780 µmol photons m^−2^ s^−1^. The spectral composition of the light with the main peaks at 540 and 610 nm and smaller peaks at 435 and 490 nm was measured by a BTS256-PAR BiTec Sensor Agriculture Light Meter (Gigahertz-Optik GmbH, Türkenfeld, Germany) and is shown in Figure S1. Algal suspensions in the photobioreactors were supplied with a gas mixture of air and CO_2_ (2% *v*/*v*), at a flow rate of 15 L h^−1^. The experiments were carried out in a batch culture regime, over a temperature range of 25–40 °C and mean irradiance of 110–750 µmol photons m^−2^ s^−1^. All experiments were conducted in triplicate.

### 2.2. The Synchronization Procedure

The cells of *G. sulphuraria* from a 3-week-old agar plate were used to inoculate the batch culture that was to be synchronized. The synchronization itself was carried out by alternating light/dark periods, the lengths of which were chosen according to the growth parameters of the cells. For the first two or three cycles, the culture was observed by light microscopy to set the correct length for both the light and dark periods. The optimal time for darkening the cells was when they started their first protoplast fission. The length of the dark period was chosen to allow all cells of the population to release their daughter cells. Once the culture was synchronous, the length of light and dark periods were kept constant. For the cell cycle experiment, a synchronized culture was diluted at the beginning of the light period to the initial dry matter (DM) concentration of 100 µg mL^−1^. The light/dark (L/D) cycle under optimal conditions was set as 16/8 h L/D.

### 2.3. Measurement of Light Irradiance

For light measurement in the cultivation cylinders, a ULM-500 universal light meter (Walz) equipped with a microsphere sensor (SQS/L, Walz, Effeltrich, Germany) was used. During the measurement, the sensor was placed directly inside the suspension in the middle of the cultivation cylinders. For light measurement of the flat photobioreactors, a quantum/radiometer-photometer (LI-COR, Inc., Lincoln, NE, USA) equipped with flat quantum sensor was used. The mean irradiance was calculated from the incident and transmitted light intensity according to the Lambert Beer law. The adjustment of irradiance was achieved by dimmable fluorescent tubes.

### 2.4. Dry Matter Determination

Dry matter was determined from 5 mL of algal suspension centrifuged at 2100× *g* for 3 min in dried and pre-weighed 5-mL test tubes. The pellet was dried at 105 °C for 12 h and weighed on a Sartorius TE214S-0CE analytical balance (Sartorius, Göttingen, Germany) [64]. Data in graphs are presented as means of 3 experiments.

### 2.5. Commitment Points, Nuclear and Cellular Division Evaluation

To determine when the cells were committed to divide, 10 mL samples were taken every 2 h, incubated in the dark at 40 °C and aerated with 2% CO_2_ (*v*/*v*) in air. Under these conditions every cell eventually divided if it had passed the commitment point [65]. About 30 h after the beginning of the light period, the number of daughter cells released were counted. By this method, the percentage of cells that had divided into 2 or 4 daughter cells was determined [34]. For routine nuclei counting, the cells were stained with 2.5 µg mL^−1^ of 4′,6-diamidino-2-phenylindole dihydrochloride (DAPI) in S buffer (0.25% (*w*/*v*) sucrose, 1 mM EDTA, 0.6 mM spermidine, 0.05% (*v*/*v*) mercaptoethanol, 10 mM Tris.HCl, pH 7.6) according to Zachleder and Cepák [66] and the nuclei were visualized by fluorescence microscopy. The filter block U-MWIBA2 (excitation/emission: 460–490/510–550 nm) was used in an Olympus BX51 microscope (Olympus, Tokyo, Japan). The proportions of mother cells and daughter cells were determined by light microscopy in cells fixed in Lugol solution (1 g I, 5 g KI, 100 mL H_2_O) at a final concentration 10 μL of Lugol solution per 1 mL of cell suspension. The sigmoidal commitment curves, nuclear division and cell division curves were obtained by plotting the cumulative percentages as a function of sampling time [45,51].

For visualization of nuclear and cell division by confocal microscopy, the cells were stained with SYBR Green according to Vítová, et al. [67], for details see also Hlavová, Vítová and Bišová [51]. In short, five microliters of freshly defrosted cell pellet were combined with 2.5 µL of SYBR Green I (ThermoFisher Scientific, Linz, Austria), vortexed and kept for 5–10 min in the dark at room temperature. Next, the cells were observed under a confocal microscope LSM Zeiss 880 (Carl Zeiss, Jena, Germany) using the following excitation wavelengths: chlorophyll—633 nm, and SYBR Green I—405 nm.

### 2.6. Cryo-Scanning Electron Microscopy (Cryo-SEM)

For cryo-SEM, the cells were transferred to holes within the SEM holder and the holder was immediately frozen in liquid N_2_ slush (CryoAlto 2500). Frozen samples were transferred into a preparative chamber (CryoAlto 2500) where they were fractured with a scalpel at −135 °C. Specimens were either without sublimation or sublimed for a short time (after the temperature reached −98 °C, the temperature was immediately decreased to −135 °C). The samples were sputter-coated with gold for 30 s. Samples were examined by JEOL 7401F SEM (Jeol Company, Tokyo, Japan) at 1.5 kV and at −135 °C using the Everhart—Thornley detector of secondary electrons.

### 2.7. RNA, DNA, Proteins Analyses

Total nucleic acids were extracted according to Wanka [68] as modified by Lukavský, et al. [69]. The samples were centrifuged in 10 mL centrifuge tubes, which also served for storage of the samples. The algal cell pellets were stored under 1 mL of ethanol at −20 °C. The algae were extracted 5 times with 0.2 M perchloric acid in 50% ethanol for 50 min at 20 °C and 3 times with an ethanol-ether mixture (3:1) at 70 °C for 10 min. Such pre-extracted samples can be stored in ethanol at −20 °C. Total nucleic acids were extracted and hydrolyzed with 0.5 M perchloric acid at 60 °C for 5 h. After hydrolysis, concentrated perchloric acid was added to achieve a final concentration of 1 M perchloric acid in the sample. The absorbance of total nucleic acids in the supernatant was measured spectrophotometrically at 260 nm (A_260_).

Light activated reaction of diphenylamine with hydrolyzed DNA, as described by Decallonne and Weyns [70] was used with the following modification [71]: The diphenylamine reagent (4% diphenylamine in glacial acetic acid, *w*/*v*) was mixed with the samples of total nucleic acid extracts in the ratio 1:1 and the mixtures in the test tubes were illuminated from two sides with fluorescent lamps (Tesla Z, 40 W, Tesla lighting, Praha, Czech Republic). The incident radiation from each side was 150 μmol photons m^−2^ s^−1^. After 6 h of illumination at 40 °C, the difference between the A_600_ and A_700_ nm was estimated. The RNA content was calculated from the difference between total nucleic acids and DNA content.

The sediment remaining after nucleic acid extraction was used for protein determination. It was hydrolyzed with 1M NaOH for 1 h at 70 °C. The protein concentration was quantified using Folin and Ciocalteu’s phenol reagent (Sigma-Aldrich, Taufkirchen, Germany) according to the procedure described by Lowry et al. [72]. The same procedure was carried out to establish a calibration curve set by different concentrations of bovine serum albumin.

### 2.8. Glycogen Analysis

Glycogen was determined according to Brányiková et al. [63]. Samples (10 mL) of algal suspension were harvested by centrifugation at 2100 g for 3 min, and the pellets were frozen at −20 °C. Frozen cells were disintegrated by vortexing (Vortex Genie 2, Scientific Industries, Inc., Bohemia, NY, USA) with 500 µL of glass beads (0.4–0.6 mm) (P-LAB, Prague, Czech Republic) for 5 min in 250 µL of distilled water. The pigments were extracted three times using 4 mL of 80% ethanol for 15 min at 68 °C. For total hydrolysis of glycogen, 3.3 mL of 30% perchloric acid were added to the sediment, stirred for 15 min at 25 °C and centrifuged. This procedure was repeated three times. The extracts were combined and made up to 10 mL. Thereafter, 500 µL of the extract were cooled to 0 °C; 2.5 mL of anthrone solution (2 g of anthrone in 1 L of 72% (*v*/*v*) H_2_SO_4_) were added and stirred. The mixture was incubated in a water bath at 100 °C for 8 min. It was then cooled to 20 °C, and the A_625_ was measured spectrophotometrically (UV-1800, Shimadzu, Kyoto, Japan). Calibration was carried out simultaneously using glucose as the standard. The values measured for glucose were multiplied by 0.9 to obtain a calibration curve for glycogen determination.

## 3. Results 

### 3.1. Optimization of Growth Conditions

To establish the optimal growth conditions for *G. sulphuraria* before synchronization experiments, the strain was cultivated autotrophically under continuous light. In the case of a mixotrophic regime, it was cultivated with the addition of 1% *v*/*v* glycerol to evaluate and optimize growth conditions of asynchronous cultures for potential biotechnological applications. Different light intensities, temperatures and pH were tested.

#### 3.1.1. Effect of Light Intensity and Temperature

**Figure 1:**
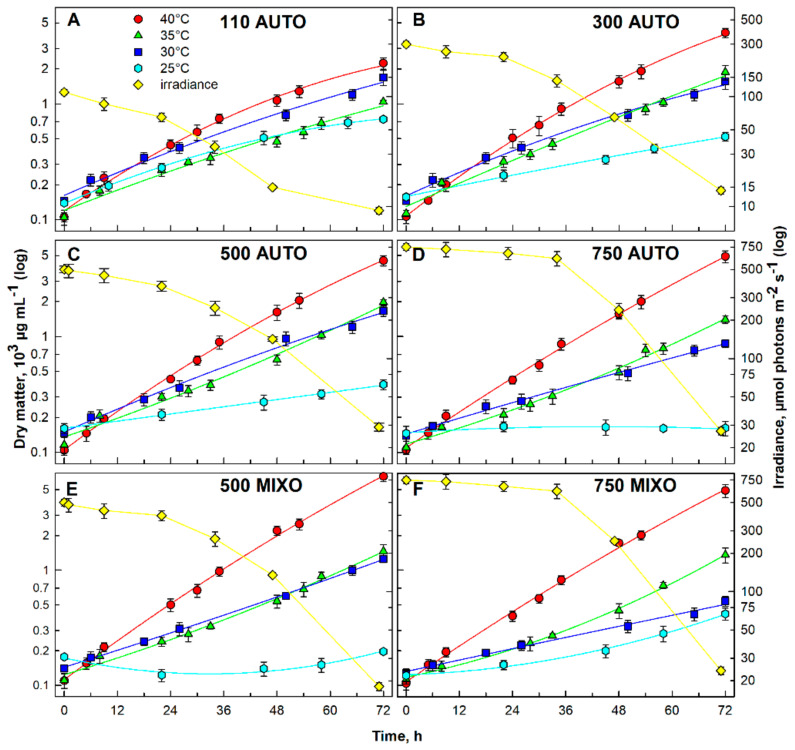
The growth of the *Galdieria sulphuraria* under auto- (panels (**A**–**D**), AUTO) and mixotrophic (panels (**E**,**F**), MIXO) regimes at different starting irradiances in the center of the cultivation cylinder (110, 300, 500 and 750 µmol photons m^−2^ s^−1^, indicated in each panel) and different temperatures (40 °C-red circles, 35 °C-green triangles, 30 °C-dark blue squares, and 25 °C-cyan hexagons). The irradiance in the center of cultivation cylinders declining with increasing cell density during the cultivation is indicated in each panel (yellow diamonds). Growth is expressed as dry matter (DM) in µg mL^−1^. The data are plotted as means of biological triplicates. The error bars represent standard deviations (±SD) and are shown when larger than the symbol size.

Four different irradiances of the starting culture in the center of the cultivation cylinder (110, 300, 500 and 750 µmol photons m^−2^ s^−1^) and four different temperatures (25, 30, 35 and 40 °C) were followed (Figure 1A–D, or Figure 1E,F, respectively). Together with the growth curves, changes in the irradiance in the center of the cultivation cylinder during cultivation at 40 °C are shown in the Figure 1 (yellow diamonds in all panels). The final dry matter (DM) ±SD values reached under the different conditions are listed in Table 1. The optimal temperature for *G. sulphuraria* was 40 °C at all mean irradiances used (Figure 1, red circles in all panels). The maximal dry matter (DM), 4.90 × 10^3^ µg mL^−1^, was reached at 40 °C and the irradiance of 750 µmol photons m^−2^ s^−1^ under an autotrophic regime (Figure 1D; Table 1). With decreasing light availability, the maximal biomass yield of autotrophic *G. sulphuraria* at 40 °C decreased (Figure 1, compare panels A–D; Table 1) to the lowest DM reached of 2.24 × 10^3^ µg mL^−1^ at 110 µmol photons m^−2^ s^−1^ (Figure 1A, Table 1). At lower temperatures (35, 30 and 25 °C) (Figure 1A–D, green triangles, dark blue squares and cyan hexagons, respectively; Table 1) growth was slower when compared to 40 °C. The temperature of 25 °C was even lethal at the highest irradiance of 750 µmol photons m^−2^ s^−1^ (Figure 1D, cyan hexagons). The best growth at 25 °C was observed at the lowest irradiance of 110 µmol photons m^−2^ s^−1^ (DM 0.73 × 10^3^ µg mL^−1^) (Table 1). A similar trend, as in the autotrophic cultures, was observed in cultures cultivated mixotrophically with the addition of 1% glycerol (Figure 1E,F), with maximum growth (DM 6.50 × 10^3^ µg mL^−1^) at 40 °C and 500 µmol photons m^−2^ s^−1^ (Figure 1E, red circles, Table 1).

The temperature of 40 °C and the starting irradiance of 500 µmol photons m^−2^ s^−1^ were chosen as the optimal conditions for further autotrophic cultivation and synchronization. An irradiance of 500 µmol photons m^−2^ s^−1^ was chosen as optimal because it is high enough for good synchronization of algae and concurrently, DM was the second highest in the autotrophic cultures; the highest DM in mixotrophic cultures was at 500 µmol photons m^−2^ s^−1^.

#### 3.1.2. Effect of pH under Different Trophic Regimes

**Figure 2:**
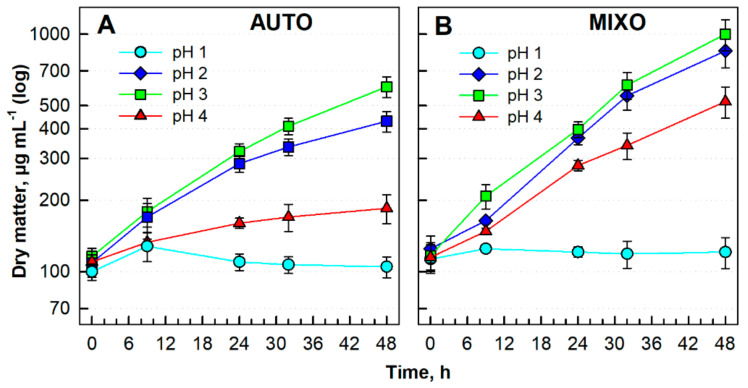
Growth of *Galdieria sulphuraria* under auto- (**A**) and mixotrophic (**B**) regimes at different pH (pH 1-cyan circles, pH 2-blue diamonds, pH 3-green squares, pH 4-red triangles), at a light intensity of 500 µmol photons m^−2^ s^−1^ and temperature of 40 °C, expressed as dry matter (DM) in µg mL^−1^. For mixotrophic growth, 1% glycerol was added. The data are plotted as means of biological triplicates. The error bars represent standard deviations (±SD) and are shown when larger than the symbol size.

To evaluate the effect of pH, the strain was cultivated under continuous light at a starting irradiance of 500 μmol photons m^−2^ s^−1^ at 40 °C under different trophic regimes for 48 h (Figure 2). The same initial cell density (dry matter) of about 100 µg mL^−1^ was applied at the beginning of the experiment to all cultures. The mixotrophic cultures grew with the addition of 1% glycerol. Comparing the autotrophic growth of *G. sulphuraria* at pH 1, 2, 3 and 4, the best growth (up to DM 600 µg mL^−1^) was reached at pH 3 (Figure 2A). Similar patterns were obtained under the mixotrophic regime where the culture reached the maximum DM of 1000 µg mL^−1^ at pH 3 (Figure 2B). Growth under mixotrophic conditions was increased under all pH conditions when compared with the autotrophic cultures. pH 1 was lethal for both cultures (Figure 2; cyan circles). pH 3 was chosen as the optimal one for further experiments.

#### 3.1.3. Synchronized Cultures

**Figure 3:**
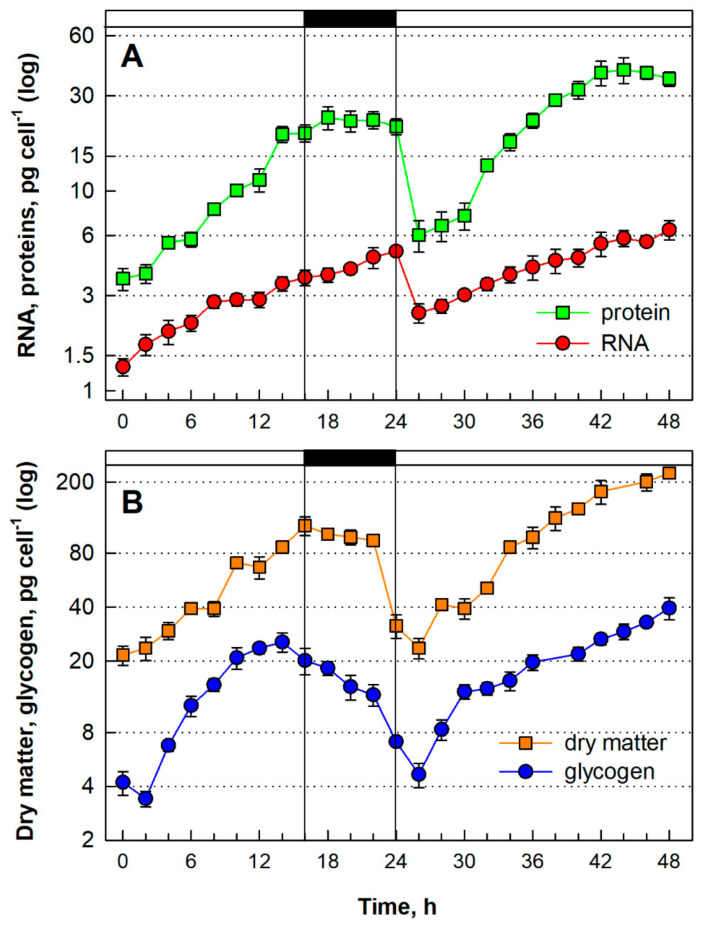
Synthesis of RNA and protein (**A**), concentration of dry matter and total glycogen (**B**) in a synchronous culture of *Galdieria sulphuraria* grown at 500 µmol photons m^−2^ s^−1^, 40 °C, pH 3. Panel (**A**): Red circles-RNA synthesis, green squares-protein synthesis. Panel (**B**): Orange squares-dry matter, blue circles-glycogen. Note the logarithmic scale on the Y axis. Horizontal dashed lines indicate doublings of the initial values. The dark phase is indicated by the black bar. The data are plotted as means of biological triplicates. The error bars represent standard deviations (±SD) and are shown when larger than the symbol size.

The established optimal conditions were used as the start point for the synchronization experiments. For the first two or three cycles, the culture was regularly observed by light microscopy to set the correct length for both the light and dark periods. Based on the culture behavior, the L/D cycle was set at 16/8 h, which was then used throughout the experiments. For the experiments, a synchronized culture was diluted to an initial DM of 100 µg mL^−1^ (Figure 3B, orange squares, calculated in the graph as 21.67 pg cell^−1^) and cultivated at a mean irradiance of 500 μmol photons m^−2^ s^−1^, 40 °C, pH 3 and L/D cycle 16/8 h for one cell cycle. Once the first cell cycle was completed, the culture was diluted to approximately the same initial concentration of 100 µg mL^−1^ and followed for another cell cycle in continuous light to let it reach a maximal DM.

#### 3.1.4. Growth and Energy Reserves Accumulation

**Figure 4:**
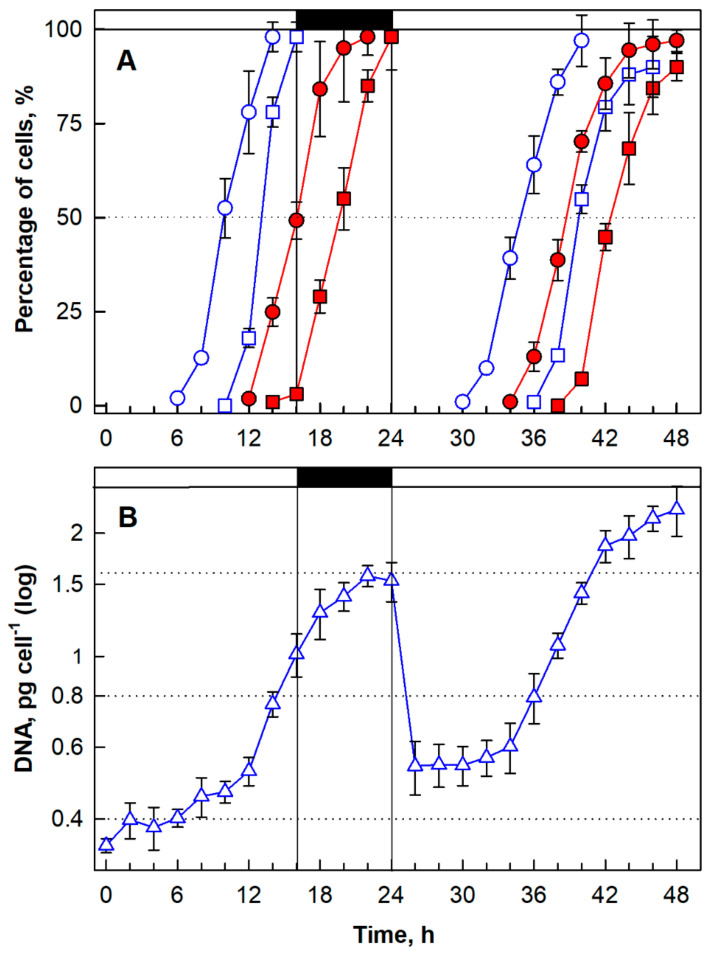
Cell cycle progression in a synchronized culture of *Galdieria sulphuraria* grown at 500 µmol photons m^−2^ s^−1^, 40 °C, pH 3. Panel (**A**): Blue lines: percentage of cells that attained the commitment point for the first (circles) and second (squares) sequence of reproductive events. Red lines: Percentage of cells that completed the first (circles) and second (squares) nuclear and cellular divisions. Cells divided into 4 daughter cells. Pane (**B**): DNA synthesis in pg cell^−1^. Dark phase is indicated by the black bar. The data are plotted as means of biological triplicates. The error bars represent standard deviations (±SD) and are shown when they are larger than the symbol size.

Growth of the synchronized culture was followed by analyzing changes in several growth parameters: total RNA, total protein, and DM. The total RNA content per cell doubled twice from the starting value in two steps at approximately the 6th and 12th hs (Figure 3A, red circles). Protein synthesis (Figure 3A, green squares) and the increase in DM (Figure 3B, orange squares) followed a similar pattern. The two maxima of all the growth parameters temporally preceded attainment of the first and second CPs at the 10th and 13th hs, respectively (Figure 4A, blue circles, blue squares, respectively). After dilution of the culture (Figure 3, from 24 h on), all growth parameters increased in a similar manner as in the first cell cycle but the individual growth steps were less visible. Similar to the first cell cycle, the increase in RNA and protein was slowed between 40–48 h, i.e., during the time of cell division (Figure 3A).

The accumulation of glycogen, the energy reserve of red algae, was followed within the synchronized culture (Figure 3B, blue circles). The net content of glycogen started to increase from the beginning of the cell cycle, reaching a peak (25.5 pg cell^−1^) at the 14th h. The glycogen content increased by about 6 fold and was consumed during the dark phase to a value of 7.1 pg cell^−1^ (Figure 3B, blue circles; dark phase indicated by the black bar). After dilution of the culture to the initial concentration, the glycogen pool started to grow again in the next cell cycle, in continuous light, and the cells acquired about 8.5-fold higher levels of glycogen (39.5 pg cell^−1^) at the end of the cell cycle (Figure 3B, blue circles, 48 h).

#### 3.1.5. Cell Cycle Progression

**Figure 5:**
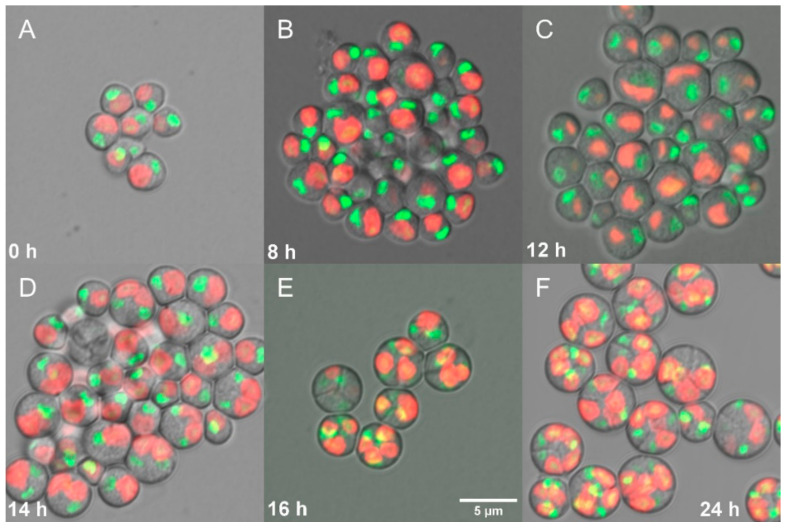
Fluorescent photomicrographs of a synchronous culture of *Galdieria sulphuraria* grown under optimal conditions (500 µmol photons m^−2^ s^−1^, 40 °C, pH 3). Daughter cells ((**A**) 0 h), growing single cells ((**B**) 8 h), chloroplast and nuclei starting to divide ((**C**) 12 h), division into 2 cells ((**D**) 14 h), division into 4 cells, protoplast division apparent ((**E**) 16 h), four daughter cells growing inside the original mother cell wall before their release ((**F**) 24 h). Nuclei in green were stained by SYBR Green I, chloroplasts in red—autofluorescence of chlorophyll. The bar represents 5 µm.

The synchronized culture attained two CPs with midpoints at about the 10_th_ and 13th hs of the cell cycle (Figure 4A, blue circles, and blue squares, respectively). Attainment of CP allowed for two rounds of DNA replication, which started by the 12th h and was completed by the 22nd h. For DNA replication, there was no visible step-wise pattern but the DNA multiplied about 4-fold as expected (Figure 4B). DNA replication was followed by nuclear division (Figure 5) and two rounds of protoplast fission with midpoints in the 15th and 19th hs (Figure 4, red circles, red squares; respectively). The daughter cells were liberated from the mother cell at the very end of the dark phase or some of them even at the beginning of the next light phase. The attainment of the first CP in the next cell cycle (34 h) was identical to attainment of the first CP in the first cell cycle with a midpoint in the 10th h (compare both lines in Figure 4A, blue circles). However, the second CP in the next cell cycle, with its midpoint in the 40th h (16th h of the second cell cycle), was delayed by 3 h when compared with the second CP in the first cell cycle, with its midpoint in the 13th h (compare both lines in Figure 4A, blue squares). DNA replication was performed similarly in both first and second cell cycles (Figure 4B). The first protoplast fission in the second cell cycle had a midpoint 2 h earlier (38 h, 14th h of the cell cycle) than the same event in the first cell cycle (compare both lines in Figure 4A, red circles). The same trend was apparent for the second protoplast fission, which was earlier in the second cell cycle by approximately 2 h (42 h, 18th h of the cell cycle) (compare both lines in Figure 4A, red squares).

**Figure 6:**
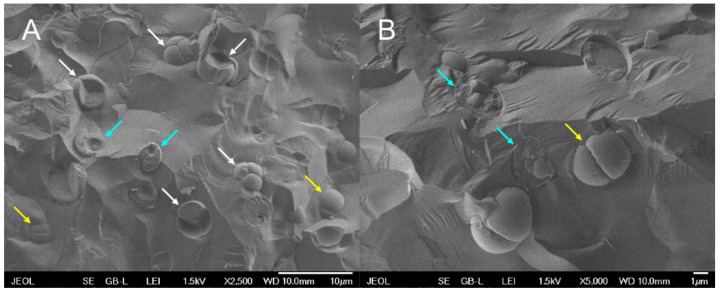
Photomicrographs of the asynchronous culture of *Galdieria sulphuraria* grown under optimal conditions (500 µmol photons m^−2^ s^−1^, 40 °C, pH 3) and visualized using a cryo-scanning electron microscope (cryo-SEM). Single daughter cells-cyan arrows, mother cells divided into 2 daughter cells-yellow arrows, mother cells divided into 4 daughter cells-white arrows (**A**,**B**). The bar represents 10 µm in (**A**) and 1 µm in (**B**).

Nuclear staining showed single small nuclei at the beginning of the cell cycle (Figure 5A) and during the growth phase (Figure 5B). Nuclear division into two started in the 12th h (Figure 5C) with 25% finished by the 14th h (Figure 5D). Nuclear division into four was visible already in the 16th h (Figure 5E). Each of the mitoses was followed by chloroplast and protoplast fissions. The cells were divided into four daughters under given conditions and the daughter cells remained inside the mother cells until the start of the next cell cycle (Figure 5F). To see all stages of the cells closely, an asynchronous culture of *G. sulphuraria* was followed by cryo-scanning electron microscopy (cryo-SEM) (Figure 6). Single daughter cells (cyan arrows), mother cells divided into 2 (yellow arrows) or 4 daughter cells (white arrows) were detected in the fractured cryo-samples (Figure 6A,B).

## 4. Discussion

For any cell cycle study, optimal growth conditions have to be determined for the model organism being used. In the case of microalgae, the main factors involved in setting the growth rates are temperature, light, pH and nutrition. As an extremophile, the red microalga *Galdieria* is well adapted to high temperatures, acidic environment and low light conditions [10,73,74]. The wide range of conditions tolerated by *Galdieria* corresponds to the geographic origin of representatives of the genus [9,75,76]. In this study, the growth conditions for *Galdieria sulphuraria* (Galdieri) Merola 002, originating from Italy, were optimized. Different irradiances of 110- to 750 μmol photons m^−2^ s^−1^ were tested under the auto- and mixotrophic regimes (Figure 1). Despite the fact that *Galdieria* is evolutionarily adapted to a low light natural environment [74,77], the two higher irradiances of 500 and 750 μmol photons m^−2^ s^−1^ led to increasing growth rates under both trophic regimes (Figure 1C–F; Table 1). This confirms the ability of *G. sulphuraria* to cope with stress caused by high light using metabolic modulation [78]. In the case of mixotrophic growth, it could also be caused by the increase in intracellular CO_2_ in *Galdieria* [79]. Interestingly, the DMs achieved were comparable under both trophic regimes at 40 °C and 750 μmol photons m^−2^ s^−1^ (Table 1), suggesting the need for further studies, particularly of photosynthetic performance. From all temperatures tested, the optimal growth temperature was 40 °C under all light conditions and trophic regimes tested (Figure 1, red circles; Table 1). It corresponds to the reported temperature optimum for *G. sulphuraria* of between 40–42 °C [10]. A temperature above 42 °C significantly reduced its growth (Figure S2), although possible tolerance of 56 °C was described in the literature [18]. Interestingly, a temperature of 25 °C was too low for efficient growth of *G. sulphuraria* (Figure 1, cyan hexagons), butit was partially compensated for under a mixotrophic regime (Figure 1F, cyan hexagons; Table 1), possibly due to metabolic modulation, allowing the cells to cope with the harsh environmental conditions [78]. From the four pH values tested, only pH 2, 3 and 4 were well tolerated by *G. sulphuraria* under both trophic regimes applied (Figure 2A,B). In contrast, pH 1 was lethal in both cultures (Figure 2, cyan circles). It was found previously that *G. sulphuraria* was adapted to an acidic pH, but changes in pH can affect the composition of the biomass [11,23]. Furthermore, pH values can differ, even for closely related species [12,16]. For further synchronization and cell cycle experiments, pH 3 was chosen. Jong et al. [43] used a similar pH value (pH 2.5) for cell cycle studies in a different strain of *G. sulphuraria*, 074W.

To study cell cycle progression, along with determining the optimal growth conditions, it is necessary to synchronize the experimental culture. In this study, the protocol for synchronization of *G. sulphuraria* was developed under optimal conditions (500 μmol photons m^−2^ s^−1^, 40 °C, pH 3). The L/D cycle was established as 16/8 h (Figure 4). Applying this L/D cycle, division into 4 daughter cells was completed within 24 h in the whole population (Figure 4, red squares). This fits with division of *G. sulphuraria* strain 074W into mostly 4 or 8 and rarely 16 daughter cells [43]. Other *Galdieria* species divide into 32 autospores [42] or 16 autospores [80]. The division number might be dependent on both the strain and culture conditions used. The daughter cells of *G. sulphuraria* stayed in the mother cell wall through the dark phase (Figure 5F), and were then liberated at the beginning of the next light phase, which is in line with the observation of *G. sulphuraria* strain 074W [43].

The synchronous culture of *G. sulphuraria* cultivated under optimal conditions increased total RNA, total protein and DM four-fold in two waves of doublings (Figure 3). Such a growth pattern is common in green microalgae dividing by multiple fission [37,38,44,81], as well as in the red alga *C. merolae*. Unlike other microalgae, the red algae, including *Galdieria,* produce glycogen instead of starch as energy and carbon reserves [26]. The pattern of glycogen synthesis through the cell cycle copied the DM pattern (Figure 3B, blue circles). The glycogen was then consumed during the dark phase. The spending of glycogen for division processes was comparable between the L/D and continuous light cultivation. However, production was not limited during continuous light cultivation, leading to higher net glycogen values. While the evolution and structure of glycogen from the *G. sulphuraria* is well-characterized [27,82], and its production for biotechnology described [26,83], the accumulation of glycogen in *Galdieria* through the cell cycle has not been studied. It can only be examined in parallel with the accumulation of starch through the cell cycle of the model green algae *Chlamydomonas reinhardtii*, *Desmodesmus quadricauda* or *Parachlorella kessleri* grown under optimal conditions. The periodicity of starch content followed the L/D cycling or, in continuous light, starch was consumed during cell division [32,36,37,57].

Cell cycle entry occurs at CP, when a reproductive sequence composed of DNA replication, nuclear and cellular divisions commences. The CPs in the synchronized culture of *G. sulphuraria* had midpoints at about the 10th and 13th hs of the first cell cycle (Figure 4A, blue circles, and blue squares, respectively). The CPs of the red algae *C. merolae* and *C. caldarium* were determined with a peak in the 10th h of the cell cycle, even at a lower light intensity [43], suggesting a quite long growth pre-CP period when compared with green algae grown under comparable conditions [28,29]. Jong et al. [43] also correlated the commitment cell size and number of cell divisions in red algae by dividing by multiple fission. The commitment cell size contributed to determining the number of successive cell divisions, as was also described in volvocine green algae. The commitment size of *G. sulphuraria* was determined to be about 4–7-fold larger than the average daughter cell volume and 4-fold larger for *C. caldarium* [43].

Evaluation of photosynthetic parameters of *G. sulphuraria* affected by different trophic regimes and growth conditions, especially in the context of photo-inhibition under high light intensities, would be a relevant future research direction. For example, photosynthesis of mixotrophic *G. sulphuraria* was boosted by enhancing carboxylation activity of Rubisco and decreasing photorespiration [79]. Similarly, Rubisco was affected by different trophic regimes in *G. phlegrea* [84].

Obtaining an understanding of the optimum temperature, light intensity, pH and cultivation conditions for growth under different trophic regimes, and glycogen accumulation, are important for any further exploitation of the biotechnologically significant alga *G. sulphuraria*. All these factors affect its growth rate, production of metabolites or capacity for bioaccumulation [18,23,85]. These new findings about the cell cycle and division of *G. sulphuraria* reflects progress in our understanding of the cell cycle in red microalgae and the successful achievement of synchronization is an essential precondition for future cell cycle studies.

## Figures and Tables

**Table 1:** Effect of different temperatures and irradiances under autotrophic and mixotrophic regimes on the growth of *Galdieria sulphuraria* (dry matter (DM), 10^3^ µg mL^−1^). Cultivated 72 h.

	Irradiance (µmol photons m^−2^ s^−1^)	Autotrophy				Mixotrophy	
Temperature (°C)		110	300	500	750	500	750
25		0.73 ± 0.022	0.52 ± 0.045	0.38 ± 0.036	0.16 ± 0.022	0.19 ± 0.007	0.41 ± 0.050
30		1.69 ± 0.253	1.56 ± 0.23	1.66 ± 0.166	0.87 ± 0.069	1.25 ± 0.05	0.54 ± 0.054
35		1.04 ± 0.031	1.87 ± 0.262	1.97 ± 0.098	1.40 ± 0.098	1.46 ± 0.204	1.35 ± 0.203
40		2.24 ± 0.246	4.10 ± 0.387	4.52 ± 0.452	4.90 ± 0.588	6.50 ± 0.65	4.90 ± 0.637

## Data Availability

Not applicable.

## Supplementary Materials

The following are available online at https://www.mdpi.com/article/10.3390/biom11070939/s1, Figure S1: Spectral composition of the light source, Figure S2: Growth of *Galdieria sulphuraria* at 44 °C and 46 °C.
