# Supplementary figures and images for: Growth under Different Trophic Regimes and Synchronization of the Red Microalga Galdieria sulphuraria
Source: Biomolecules. 2021 Jun 24;11(7):939. doi: 10.3390/biom11070939 (PMC8301940; doi:10.3390/biom11070939)

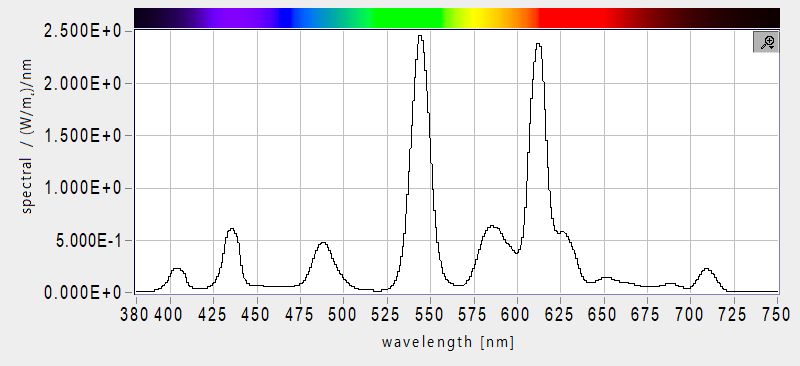

Supplement: Supplementary file 1 [file biomolecules-11-00939-s001.zip › FigS1.jpeg]

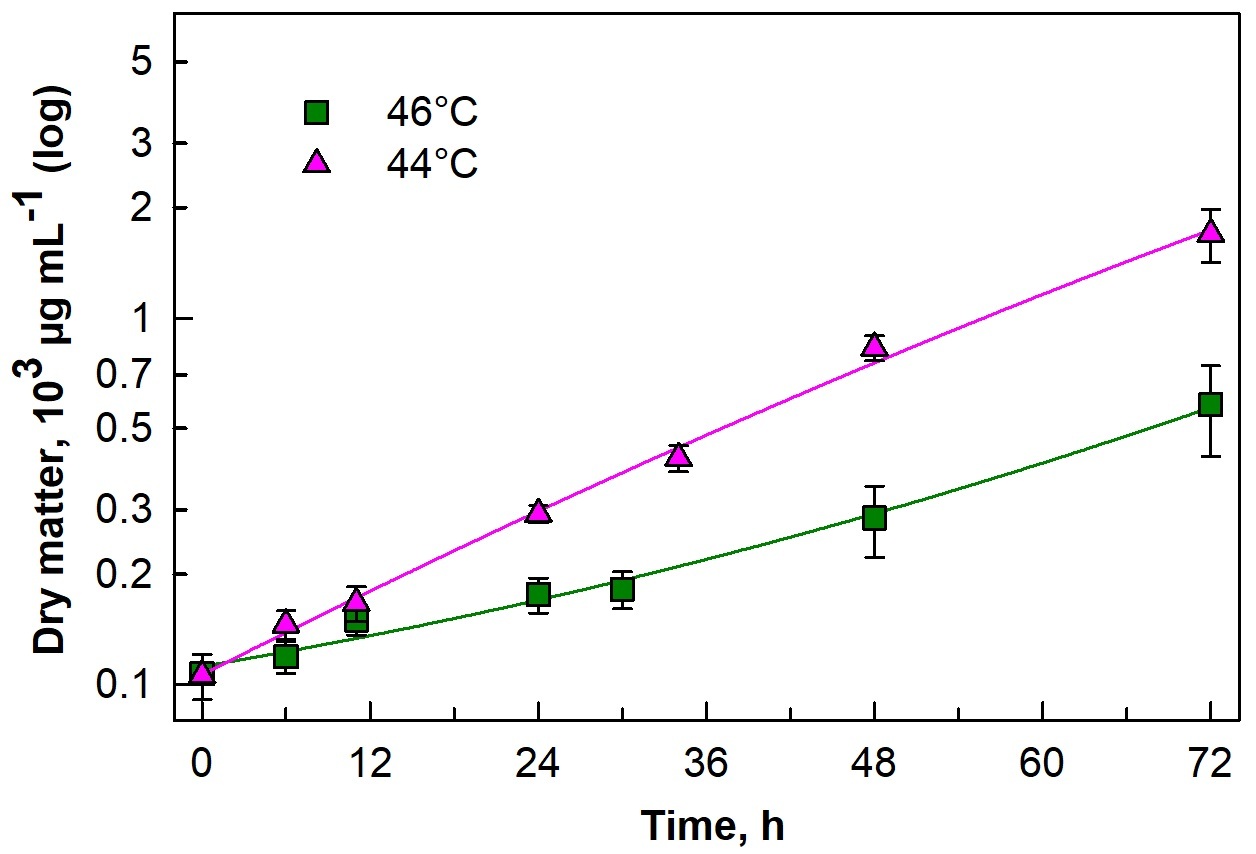

Supplement: Supplementary file 1 [file biomolecules-11-00939-s001.zip › FigS2_160621_sd.jpeg]
